# Incidence and predictors of early loss to follow up among patients initiated on protease inhibitor-based second-line antiretroviral therapy in southwestern Uganda

**DOI:** 10.1186/s12981-021-00331-5

**Published:** 2021-03-20

**Authors:** Edwin Nuwagira, Boniface A. E. Lumori, Rose Muhindo, Michael Kanyesigye, Abdallah Amir, Winnie Muyindike, Conrad Muzoora

**Affiliations:** 1 Mbarara University of Science and Technology, P.O Box 1410, Mbarara, Uganda; 2 Immunosuppression Clinic Mbarara Regional Referral Hospital, Mbarara, Uganda

**Keywords:** HIV, Second-line antiretroviral therapy, Uganda, Loss to follow-up, Protease inhibitors

## Abstract

**Background:**

Good adherence to antiretroviral therapy (ART) and retention in care are essential for the effectiveness of an HIV care program. With the current increase in numbers of people living with HIV taking second-line ART in sub-Saharan Africa, there is a need to establish their treatment outcomes and the rate of loss to follow up. In this study, we determined the incidence and predictors of loss to follow up among patients taking second-line ART at an experienced HIV treatment center in southwestern Uganda.

**Methods:**

This was a retrospective review of an electronic database at Mbarara Regional Referral Hospital HIV clinic in southwestern Uganda. Second-line ART included at least two of the nucleoside reverse transcriptase inhibitors and a boosted protease inhibitor. Loss to follow-up was defined as failure to return to the health facility for care or treatment refill for 180 days or more from the previous visit. After excluding children less than 15 years, we pooled data that included socio-demographic, clinical, and laboratory data for patients who started second-line ART between 2002 and 2017. Multiple imputation was done for variables with missing data. Variables that had a p < 0.05 in unadjusted bivariate analyses were included in a multivariate binomial regression model using a stepwise backward selection procedure to describe the factors that independently predicted loss to follow-up.

**Results:**

Between 2002 and 2017, 1121 patients had been initiated on second-line ART. We included data from 924 participants and of these, 518 (56.1%) were female, the mean age (SD) was 38.4 (± 10.5) years, and 433 (52.4%) had a CD4 count less than 100 cells/µl at the start of second-line ART. The incidence of loss to follow-up was 26.7 per 100 person-years. Male gender (Adjusted risk ratio (ARR) = 1.8, 95% CI 1.5–2.0) p < 0.001 and anemia ARR 1.4, 95% CI 1.1–1.6) p < 0.001 were strongly associated with loss to follow up.

**Conclusions:**

There is a high incidence of loss to follow up among patients taking protease-inhibitor based second-line ART at a tertiary HIV center in southwestern Uganda. There is a need to routinely measure hemoglobin during clinic reviews, and establish mechanisms to retain males initiated on second-line ART in care. The association of anemia and loss to follow up needs to be investigated.

## Introduction

The global prevalence of HIV is declining with the majority of new infections due to the unmet HIV control measures among the key populations [1, 2]. By the end of June 2020, approximately 26 million people living with HIV (PLWH) were on antiretroviral therapy (ART) worldwide and despite the global scale-up of ART, there were an estimated a million deaths due to AIDS-related illnesses [1]. Currently, Uganda has a national HIV prevalence of 6.2% with the southwestern region having the second highest prevalence of HIV in the country at 7.9%. ART coverage is at 90.4%, and the overall viral suppression is at 59.6% [3].

Expansion of HIV viral load testing (virological monitoring) has led to early detection of treatment failure, and an increase in numbers starting on second-line ART [4]. This has resulted in an escalating need for second-line therapy which is expected to rise to three times the current state by 2030 [5]. While ART significantly improves life expectancy and reduces mortality among PLWH [6], its effectiveness is hindered by loss to follow up which is estimated to be between 25 and 62% in sub-Saharan Africa [6–9].

Studies have shown that LTFU is indeed a major challenge for ART programs in resource-limited settings because it is associated with treatment disruption, subsequent ART failure, and mortality [9, 10]. Loss to follow-up of PLWH has negative impacts on their immunological status and increases their chances of suffering from opportunistic infections which is costly to the public ART programs that are already battling limited resources. Since protease-inhibitor (PI) based second-line ART is costly and not readily available to most treatment centers in Uganda, failure on this class of drugs will increase the risk of mortality due to opportunistic infections [6, 9] or progression to the current last resort which is third-line ART that is much more expensive than second-line ART [10, 11]. Failure on potent regimens and increased demand for subsequent regimens that are costly or not available reduces the chances of achieving the last 90′ and HIV eradication by 2030. Different studies have shown that factors associated with loss to follow-up include; gender [7, 9, 12], adolescent age [13], marital status [14, 15], time and distance from the health facility [15], occupation, and low socioeconomic state [16]. These studies, however, included all patients on ART hence the need to establish factors that predict loss to follow of patients on second-line ART.

This to our knowledge has not been done in Uganda and would inform our daily clinical practice about which patients on PI-based regimens would benefit from active follow up.

We, therefore, sought to determine the one-year incidence rate of loss to follow-up, and to explore the socio-demographic factors and clinical characteristics associated with loss to follow up among patients taking second-line ART at a tertiary HIV clinic.

## Methods

### Study design, setting and population

This was a retrospective study was conducted at Mbarara Regional Referral Hospital (MRRH) which is located in the Mbarara Municipality, about 260 km from Kampala, the capital city of Uganda. MRRH serves as the teaching hospital for Mbarara University of Science and Technology and has been a model HIV case center since 2003. The clinic provides both pediatric and adult HIV care services to over 4 million people from southwestern Uganda [17]. This clinic has provided care to approximately thirty thousand patients since its inception. In this clinic, patients are counseled at the time first-line ART is initiated, followed up and further adherence counseling done before they start second-line ART. Patients starting a new ART regimen are usually given monthly drug refills for the first six months and adherence is monitored by using self-reported and electronic adherence calculation, using the pill count method. Patients’ information at this HIV clinic was previously documented only on paper but, currently, all patient information is stored in an Electronic Medical Records system (MRS) which started in 2007. All information that was previously stored in paper files had been transferred to the database. The paper files are still used as daily clinical review forms and data from these files is entered into the open MRS by qualified data personnel. The team performs routine data quality control procedures and all queries are corrected within 24 h. We pooled data, from adolescents and adults (> 15 years) living with HIV who had evidence of failure on first-line ART and had been started on second-line ART between 2007 and 2016, with a maximum follow-up of one year. Patients who were taking second-line ART but had never taken first-line ART were excluded. We also excluded patients that had been taking second-line ART, but later switched to first-line ART.

### Variables

In our study, individual patient-level data were pooled from the MRS. We were interested in socio-demographic variables like age, gender, marital status, patient category (transfer in versus original cohort), religion, level of education, and the number of dependants including children.

The clinical variables included the commonly diagnosed opportunistic infections at this clinic and these are; Tuberculosis, Kaposi’s Sarcoma, and Cryptococcal meningitis [18]. Laboratory variables included the values obtained from the complete blood count, such as the hemoglobin count and total lymphocyte counts. We included these two basing on previous studies that had identified anemia and total lymphocyte count as predictors of treatment outcomes in patients with HIV [1]. CD4 T cell count recorded had been measured using either standard flow cytometric methods (Coulter Epics Cytometer, Backman Coulter, Brea California) or a point of care PIMA-CD4 analyzer machine (Alere Waltham MA).

### Data Extraction procedures

We pooled data that included all the baseline variables of interest at the start of second-line ART. Since our HIV-clinic data is an open MRS database, we integrated Structured Query Language (SQL) in STATA version 15 to run over the database and compile patients who were on PI-based second-line ART and above 15 years. These were then transferred to a separate STATA (Stata Corp, College Station, Texas, USA) do-file. We again used the SQL language to generate variables which we needed to have a cleaner do-file with only participants that were meeting our inclusion criteria.

### Definition of endpoints

The primary outcome was loss to follow-up. Switch to second-line ART was defined as changing at least one of the nucleoside reverse transcriptase inhibitors in the initial first-line regimen and adding a protease inhibitor to the regimen. Our time zero was the date of initiation of second-line ART. We evaluated the first 12 months of the patients on second line ART, where day-zero was the first day of starting a P.I-based regimen. Loss to follow-up in this study was defined as failure to return to the health facility for care or treatment refill for a period of 180 days basing on the previous definitions of loss to follow-up in an HIV-infected population [20].

“Transfer-out” patients were those who had been documented as transferred from the MRRH-HIV clinic to another HIV treatment facility, whereas death was defined as the recorded death in the first year of taking second-line ART.

### Ethical consideration

This work was done after acquiring all the necessary ethical approval from the Mbarara University of Science and Technology (MUST) research and ethics committee, the MRRH Director, and the Uganda National Council of Science and Technology (UNCST). We received a waiver of consent since this was a retrospective review of patients’ records. All data were de-identified and the MRRH HIV clinic identification numbers were replaced with our study-generated identification numbers.

### Analysis

Since the data was generated into Stata version 15 during extraction, we used the same software for analysis. Categorical variables were expressed as proportions whereas continuous variables were expressed as means with a standard deviation if normally distributed and, median with an inter-quartile range for skewed data to describe sociodemographic, clinical and laboratory characteristics at the start of second-line ART.

For the incidence rate, we calculated every participant’s person-time in years, and parameters were given a 95% confidence interval for reliability of our results. The cumulative incidence was calculated as a percentage where, the numerator was the patients who were lost to follow-up within 12 months from the day of starting P.I-based second-line ART, whereas the denominator included all those who had been switched to a second-line ART regimen and completed at least one year on second-line ART (Including those lost to follow up, dead, transferred out and those active in care). We used the baseline demographics, clinical examination findings and laboratory values like CD4, hemoglobin and total lymphocyte count to generate factors associated with LTF using a binomial regression model.

Because we used secondary data, we had variables with missing data. We used the *mdesc* command to establish the proportion of missing data for each variable. Variables that had more than 30% of missing data were not included in the regression model.

We assumed that the missing data was missing at random (MAR), since it had been collected on paper files prior to being entered in the database. Also, the clinical case forms had been updated over the years with introduction of new variables that were missing in the old cohort of patients. We multiply imputed missing values using variables with missing data with those that had complete data [21]. We identified potential auxiliary variables and included them in our imputation model to improve the quality of all imputed variables [22]. Since we had a sufficient sample size, we implored the data augmentation (DA) algorithm of the Markov Chain Monte Carlo (MCMC) procedure to fill in missing data [23]. Imputed procedures used chained equations with 10 repetitions [24].

In a binomial regression model, we set up the analysis so that for each variable, the reference category was that which we hypothesized to have the lowest risk of loss to follow up. For example, having Stage one HIV was thought to be associated with retention into care, thus used as the reference range for that category. Variables that had a P-value less than 0.05 in the bivariate analysis were included in the multivariate analysis using a binomial regression model that was performed in a backward stepwise selection method to determine significant independent predictors of loss to follow up. Adjusted risk ratios with associated 95% confidence intervals were calculated. Factors that had a p value of 0.05 were considered to be significant.

## Results

**Fig. 1:**
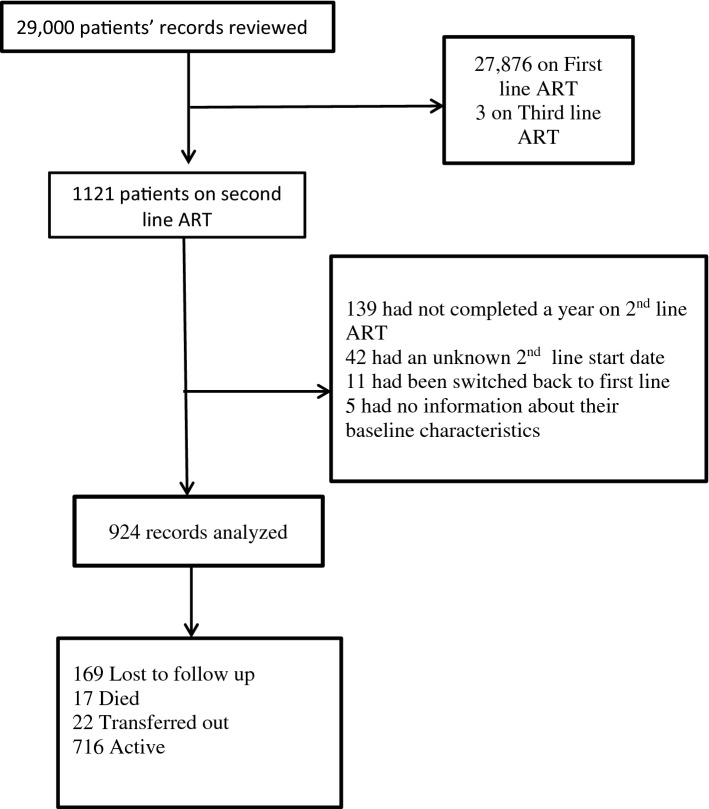
Study flow diagram

**Fig. 2:**
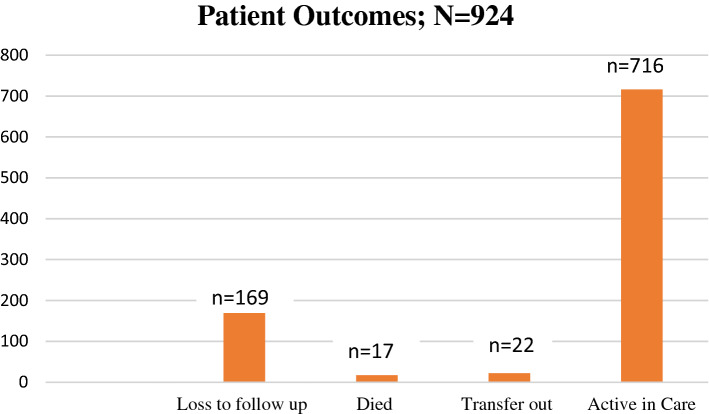
Outcomes of patients on second-line ART

Of the 1121 participants who had been initiated on second line ART since 2007, 924 (82.4%) met the study inclusion criteria (Fig. 1). Of these, 716 patients were active in care, 17 had died, 22 transferred out and 169 lost to follow-up (Fig. 2).

### Baseline characteristics

Of the participants that met our inclusion criteria, 517 (56.1%) were females, the mean age (± SD) of the included patients was 38.4 ± 10.5 years. Majority of the participants were between 35 and 49 years of age at the start of second-line ART. The median CD4 count (IQR) at starting second-line ART was 95 cells/µl (IQR 48–195) for all participants.

We created CD4 categories and 433 (52%) had CD4 count less than 100 cells/µl at the time of starting second line ART. The other baseline characteristics are displayed in Table 1.

**Table 1:** Sociodemographic, clinical and laboratory characteristics at the start of second line ART

Characteristic, N = 924	Missing n (%)	
Age in years, mean (SD)	–	38.4 ± (10.5)
Female gender n (%)	–	518 (56.1)
Transfer in	–	242 (26.2)
Baseline CD4 cells/mm^3^ median (IQR)	97 (10)	95 (48–195)
Baseline CD4 categories		
< 100	97 (10)	433 (52.4)
100–250		256 (31)
251–500		100 (12.1)
> 500		38 (4.6)
BMI in kg/m^2^ mean (SD)	240 (25.9)	21.6 (4.4)
Viral load (copies/ml) median (IQR)	483 (52.3)	35,500 (11,294–1,189,000)
Baseline lymphocyte count median (IQR)	310 (33.6)	1.2 (1–1.9)
Baseline Hemoglobin (g/dl), mean ± (SD)	149 (16.1)	12.8 ± 2.1
Switch criteria n (%)		
Clinical failure	96 (10.4)	256 (30.9)
Immunological failure		307 (37.1)
Virological failure		264 (31.9)
Marital status n (%)		
Single	58 (6)	424 (49)
Married		442 (51)
Employed n (%)	74 (8)	753 (81.8)
Self-reported Poor Adherence status, n (%)	–	799 (86.5)
Alcohol Intake, n (%)	482 (52.2)	
Yes		119 (24.7)
No		363 (75.3)
First Line ART Backbone, n (%)		
D4T or DDI based		357 (38.8)
AZT and3TC based		418 (45.4)
3TC and TDF	-	146 (15.8)
Firstline ART Start Year		
< 2006	24	444 (49.3)
2006–2010		278 (30.9)
> 2010		178 (19.8)
History of opportunistic infections		
Kaposi’s Sarcoma		33 (28.1)
Cryptococcal meningitis		10 (8.7)
Tuberculosis	–	73 (63.2)
HIV staging		
1	56 (6.1)	382 (44)
2		177 (20.4)
3		195 (22.5)
4		114 (13.1)

### Loss to follow up

The incidence rate of loss to follow up one year after switching to a second-line ART regimen was 26.7 per 100 person-years after a total follow up time of 617 years. One hundred and sixty-nine (169) 18.3% of the 924 participants enrolled in our study were lost to follow up.

### Factors associated with loss to follow up

In bivariate analysis, variables that had a p-value of less than 0.05 were included in a multivariate binomial regression model as shown in Table 2. In multivariate analysis, Males had an 80% risk of dropping out of care when compared to females (adjusted risk ratio, 1.8; 95% C.I, 1.5–2.0 p < 0.001), and in patients with anemia (hemoglobin level less than 12 g/dl for males and less than 13 g/dl for females), the risk of loss to follow up was 40% (adjusted risk ratio, 1.4; 95% C.I, 1.1–1.6 p < 0.001). Patients who started ART after 2006 were more likely to be retained into care (adjusted risk ratio 0.6; 95% C.I, 0.5.0.7 p < 0.001). Despite the wide confidence intervals, patients with a normal and high body mass index were more likely to stay in care compared to those that were underweight (Table 2).

**Table 2:** Factors associated with loss to follow up among patients initiated on second-line ART at MRRH HIV Clinic

Characteristic	Active n (%)	LTFU n (%)	Unadjusted risk ratio [C I]	p values	Adjusted Risk Ratios [C.I]	p values
Male gender	324 (79.8)	82 (20.2)	1.1 [1.02–1.2]	0.01	1.8 [1.5–2.0]	< 0.001
Age categories						
18–24	52 (77.6)	15 (33.4)	1			
25–34	225 (82.7)	47 (17.3)	0.9 [0.8–1.1]	0.43		
35–49	398 (83.6)	78 (16.4)	0.9 [0.7–1.1]	0.19		
≥ 50	80 (73.4)	29 (26.1)	1.3 [1.2–1.6]	< 0.001		
Patient category						
Original cohort	587 (86.1)	95 (13.9)	1			
Transfer in	168 (69.4)	74 (30.6)	1.9 [1.8–2.1]	< 0.001	0.9 [0.8–1.2]	0.62
CD4 categories						
< 100	371 (85.7)	62 (14.3)	1			
100–250	219 (85.6)	37 (14.4)	1 [0.9–1.41]	0.63		
251–500	86 (86)	14 (14)	1 [0.9–1.3]	0.38		
> 500	35 (92.1)	3 (7.9)	0.4 [0.3–0.6]	< 0.001		
Body-mass index Underweight						
Normal BMI	115 (78.8)	31 (21.2)	1			
Overweight	390 (87.8)	54 (12.2)	0.6 [0.5–0.7]	< 0.01	0.8 [0.7–1.0]	0.03
Obese	59 (90.8)	6 (9.2)	0.4 [0.3–0.5]	< 0.01	0.7 [0.5–1.0]	0.03
	25 (86.2)	4 (13.8)	0.3 [0.2–0.6]	< 0.01	0.2 [0.05–0.5]	0.001
Viral load						
< 1000	12 (80)	3 (20)	1			
> 1000	390 (91.2)	39 (8.8)	0.4 [0.3–0.5]	< 0.001		
Hemoglobin						
No Anemia	422 (88.7)	54 (11.3)	1			
Mild Anemia	138 (85.2)	24 (14.8)	1.3 [1.1–1.5]	< 0.001	1.4 [1.1–1.6]	< 0.001
Moderate- Severe Anemia	111 (81)	26 (19)	1.8 (1.5–2.0)	< 0.001	1.4 [1.1–1.7]	< 0.001
Marital status						
Married	342 (80.1)	82 (19.3)	1			
Single/widowed/	364 (82.3)	78 (17.7)	0.9 (09–1.1)	0.84		
Divorced						
HIV staging						
1	334 (87.4)	48 (12.6)	1		1	
2	152 (85.9)	25 (14.1)	1.1 [0.6–1.5]	0.09	1.2 [1.0–1.4]	0.04
3	165 (84.6)	30 (15.4)	1.2 [1.1–1.4]	0.06	0.9 [0.7–1.1]	0.22
4	91 (79.8)	23 (20.2)	1.3 [1.1–1.5]	< 0.01	0.7 [0.5–1.0]	0.03
Year of Starting ART						
Before 2006	351 (79.1)	93 (20.9)	1			
2006 to 2010	233 (83.8)	45 (16.2)	0.6 [0.5–0.7]	< 0.001	3.9 [2.9–5.3]	< 0.001
2011 on wards	155 (87.1)	23 (12.9)	0.6 [0.5–0.7]	< 0.001	2.2 [1.7–3.0]	< 0.001
History of Kaposi						
Sarcoma	26 (78.8)	7 (21.2)	1.1 [0.9–21.5]	0.2		
History of Tuberculosis	60 (82.2)	13 (17.8)	0.91 [0.7–1.0]	0.14		
Switched due to Clinical Failure^a^	182 (71.1)	74 (28.9)	2.2 (2.1–2.4)	< 0.00		

The Body Mass Index is the weight in kilograms divided by the square of the height in meters

^a^Clinical failure due to other opportunistic diseases other than Kaposi’s Sarcoma, Tuberculosis and Cryptococcal meningitis

## Discussion

In developing countries, loss to follow-up is a major setback to treatment success and complicates the evaluation of an HIV care program. This study sought to determine the incidence and predictors of early loss to follow-up in patients taking PI-based second-line ART. We found that the incidence of loss to follow-up was 26.7 per 100 person-years and the factors associated with LTF were; male gender, anemia (hemoglobin level less than 12 g/dl for males and less than 13 g/dl for females), and history of cryptococcal meningitis at the start of second-line ART. These are high figures compared to a South African cohort study where the loss to follow up in one year among patients on second line ART was 10.2% [25]. In Wakiso, central Uganda, the loss to follow-up rate at a small HIV clinic was 21 per 1000 person-years [16]. The differences in the incidence may be due to the differences in facility setting. In central Uganda, which is also the business district of Uganda, majority of the HIV treatment clinics are centers of excellence where patients easily access the health facility, have good follow-up mechanisms and often have treatment partners that support drug adherence. Our study was conducted at a tertiary center that serves a large population in rural southwestern-Uganda, where patients travel for long distances to access care. It is possible that patients who fail on first-line, are also likely to fail on second-line ART as well if the causes of failure such as poor adherence are not addressed. If these patients continue attending clinics that are already overwhelmed by numbers, they are likely to be lost to follow up in the first year of starting second-line ART [16, 25]. More so, this is a generally a young population starting on second-line therapy is most likely to face adherence challenges, also known as “treatment fatigue” [26, 27].hence the high risk of loss to follow up.

Similarly, other studies from developing countries have shown that males [16, 26, 28] with advanced HIV disease (low CD4) [26, 29–31] and low body mass index [32] are likely to be lost to follow up. In our study, males were indeed more likely to be lost to follow-up compared to women. It is possible that males are less likely to seek health care, as noted in previous studies and more likely to move to other places for employment without notifying their primary health care facilities.

Our study found that patients who had started on first line ART before 2006 were less likely to be retained in care compared to those who started in later years. During this time, patients were taking Stavudine (D4T) and Zidovudine (AZT) as the backbone first-line agents before D4T was dropped by the WHO in 2009 [33]. Other studies have also shown that patients that were enrolled into care before 2008 are more likely to drop out of ART care programs in SSA [34], and that these drugs contributed to attrition [18].

A low baseline hemoglobin count was associated with loss to follow-up in our study and this confirms the findings by Asiimwe et al., a study that looked at predictors of drop out from HIV care at a public facility in SSA [18]. Our study, however, used the WHO recommended cut off values for both males and females compared to the 11 g/dl used by Asiimwe and colleagues [35]. Since not all patients had baseline hemoglobin measured, it is also possible that patients who had this test were unwell and therefore more likely to be lost to follow-up.

Our biggest limitation is the difference in the definitions of loss to follow-up and it has been noted that results of retention proportions presented are often affected by the choice of loss to follow-up definition [36]. Our study used a universally acceptable definition of loss to follow-up in HIV care programs [37], so that our results can be generalizable to other HIV treatment centers that provide second-line ART. Secondly, this was a retrospective cohort study and information bias may have occurred due to underreporting of some conditions and uncollected data, leading to missing data. However, previous studies done at our site have shown that patients with complete data were not significantly more likely to be lost to follow-up than those without data [18]. We also interpret our data to mean that it is possible that most of the people who are lost to follow-up die, since there are limited treatment options after second-line ART.

## Conclusion and recommendations

Our findings show that there is a high incidence of loss to follow-up in the first year of starting P.I-based second-line ART. The factors associated with loss to follow-up include, a history of cryptococcal meningitis at initiation of second-line ART, male gender, and anemia.

We recommend designing and validating a clinical prediction tool using the above identified factors for easy identification of patients on second-line ART who would need active follow-up, to improve retention of patients on second-line ART in our HIV treatment program. We also recommend active follow-up of these patients initially lost to follow-up to ascertain their true outcomes.

## Data Availability

The datasets used during the current study are available from the corresponding author on reasonable request.

## Abbreviations

ART: Antiretroviral therapy
AZT: Zidovudine
D4T: Stavudine
MRRH: Mbarara Regional Referral Hospital
PI: Protease inhibitor
PLWH: People living with HIV
SSA: Sub Saharan Africa
WHO: World Health Organization

## References

[1] UNAIDS. Global HIV & AIDS statistics — 2020 fact sheet. 2020. p. 1. (GLOBAL HIV STATISTICS). https://www.unaids.org/en/resources/fact-sheet. Accessed 31 Aug 2020.

[2] Stannah J, Dale E, Elmes J, Staunton R, Beyrer C, Mitchell KM (2019). HIV testing and engagement with the HIV treatment cascade among men who have sex with men in Africa: a systematic review and meta-analysis. Lancet HIV.

[3] Ministry of Health, Uganda. UGANDA POPULATION-BASED HIV IMPACT ASSESSMENT UPHIA 2016–2017. 2018 Apr. https://phia.icap.columbia.edu/wp-content/uploads/2018/07/3430•PHIA-Uganda-SS_NEW.v14.pdf

[4] Haas AD, Keiser O, Balestre E, Brown S, Bissagnene E, Chimbetete C (2015). Monitoring and switching of first-line antiretroviral therapy in adult treatment cohorts in sub-Saharan Africa: collaborative analysis. Lancet HIV.

[5] Estill J, Ford N, Salazar-Vizcaya L, Haas AD, Blaser N, Habiyambere V (2016). The need for second-line antiretroviral therapy in adults in sub-Saharan Africa up to 2030: a mathematical modelling study. Lancet HIV.

[6] May MT, Gompels M, Delpech V, Porter K, Orkin C, Kegg S (2014). Impact on life expectancy of HIV-1 positive individuals of CD4+ cell count and viral load response to antiretroviral therapy. AIDS Lond Engl.

[7] Chammartin F, Zürcher K, Keiser O, Weigel R, Chu K, Kiragga AN (2018). Outcomes of patients lost to follow-up in African antiretroviral therapy programs: individual patient data meta-analysis. Clin Infect Dis.

[8] Fuente-Soro L, López-Varela E, Augusto O, Bernardo EL, Sacoor C, Nhacolo A (2020). Loss to follow-up and opportunities for reengagement in HIV care in rural Mozambique: a prospective cohort study. Medicine (Baltimore).

[9] Seifu W, Ali W, Meresa B (2018). Predictors of loss to follow up among adult clients attending antiretroviral treatment at Karamara general hospital, Jigjiga town, Eastern Ethiopia, 2015: a retrospective cohort study. BMC Infect Dis.

[10] Ajose O, Mookerjee S, Mills EJ, Boulle A, Ford N (2012). Treatment outcomes of patients on second-line antiretroviral therapy in resource-limited settings: a systematic review and meta-analysis. AIDS.

[11] IRIN. Striving to provide first-, second- and third-line ARVs. 2020. https://www.thenewhumanitarian.org/news/2010/12/01/striving-provide-first-second-and-third-line-arvs

[12] Frijters EM, Hermans LE, Wensing AM, Devillé WL, Tempelman HA, De Wit JB (2020). Risk factors for loss to follow-up from antiretroviral therapy programmes in low-income and middle-income countries. AIDS.

[13] Kiwanuka J, Mukulu Waila J, Muhindo Kahungu M, Kitonsa J, Kiwanuka N (2020). Determinants of loss to follow-up among HIV positive patients receiving antiretroviral therapy in a test and treat setting: A retrospective cohort study in Masaka, Uganda. PLoS ONE.

[14] Meloni ST, Chang C, Chaplin B, Rawizza H, Jolayemi O, Banigbe B (2014). Time-dependent predictors of loss to follow-up in a large HIV treatment cohort in Nigeria.

[15] Bekolo CE, Webster J, Batenganya M, Sume GE, Kollo B (2013). Trends in mortality and loss to follow-up in HIV care at the Nkongsamba Regional hospital, Cameroon. BMC Res Notes.

[16] Opio D, Semitala FC, Kakeeto A, Sendaula E, Okimat P, Nakafeero B (2019). Loss to follow-up and associated factors among adult people living with HIV at public health facilities in Wakiso district, Uganda: a retrospective cohort study. BMC Health Serv Res.

[17] Ministry of Health. Mbarara Regional Referral Hospital. 2011. https://www.health.go.ug/sites/default/files/Mbarara_RRH.pdf

[18] Asiimwe SB, Kanyesigye M, Bwana B, Okello S, Muyindike W (2015). Predictors of dropout from care among HIV-infected patients initiating antiretroviral therapy at a public sector HIV treatment clinic in sub-Saharan Africa. BMC Infect Dis.

[19] Asiimwe SB, Kanyesigye M, Bwana B, Okello S, Muyindike W (2015). Predictors of dropout from care among HIV-infected patients initiating antiretroviral therapy at a public sector HIV treatment clinic in sub-Saharan Africa. BMC Infect Dis.

[20] Chi BH, Yiannoutsos CT, Westfall AO, Newman JE, Zhou J, Cesar C (2011). Universal definition of loss to follow-up in HIV treatment programs: a statistical analysis of 111 facilities in Africa, Asia, and Latin America. PLoS Med.

[21] Royston P (2004). Multiple imputation of missing values. Stata J.

[22] Young R, Johnson DR. Imputing the missing Y’s: implications for survey producers and survey users. In 2010. p. 6242–8.

[23] Demirtas H (2010). An application of multiple imputation under the two generalized parametric families. J Data Sci.

[24] Schafer JL, Olsen MK (1998). Multiple imputation for multivariate missing-data problems: A data analyst’s perspective. Multivar Behav Res.

[25] Shearer K, Evans D, Moyo F, Rohr JK, Berhanu R, Van Den Berg L (2017). Treatment outcomes of over 1000 patients on second-line, protease inhibitor-based antiretroviral therapy from four public-sector HIV treatment facilities across Johannesburg South Africa. Trop Med Int Health.

[26] Okoboi S, Ding E, Persuad S, Wangisi J, Birungi J, Shurgold S (2015). Community-based ART distribution system can effectively facilitate long-term program retention and low-rates of death and virologic failure in rural Uganda. AIDS Res Ther.

[27] Tsegaye E, Worku A (2011). Assessment of antiretroviral treatment outcome in public hospitals, South Nations Nationalities and Peoples Region Ethiopia. Ethiop J Health Dev.

[28] Assemie MA, Muchie KF, Ayele TA (2018). Incidence and predictors of loss to follow up among HIV-infected adults at Pawi General Hospital, northwest Ethiopia: competing risk regression model. BMC Res Notes.

[29] Arnesen R, Moll AP, Shenoi SV (2017). Predictors of loss to follow-up among patients on ART at a rural hospital in KwaZulu-Natal, South Africa. PLoS ONE.

[30] Claborn KR, Meier E, Miller MB, Leffingwell TR (2015). A systematic review of treatment fatigue among HIV-infected patients prescribed antiretroviral therapy. Psychol Health Med.

[31] Mekuria LA, Prins JM, Yalew AW, Sprangers MA, Nieuwkerk PT (2015). Retention in HIV care and predictors of attrition from care among HIV-infected adults receiving combination anti-retroviral therapy in Addis Ababa. PLoS ONE.

[32] Mutasa-Apollo T, Shiraishi RW, Takarinda KC, Dzangare J, Mugurungi O, Murungu J (2014). Patient retention, clinical outcomes and attrition-associated factors of HIV-infected patients enrolled in Zimbabwe’s National Antiretroviral Therapy Programme, 2007–2010. PLoS ONE.

[33] World Health Organization. Antiretroviral therapy for HIV infection in adults and adolescents: recommendations for a public health approach-2010 revision. New York: World Health Organization; 2010. 23741771

[34] Eguzo K, Lawal A, Umezurike C, Eseigbe C (2015). Predictors of loss to follow-up among HIV-infected Patients in a rural south-eastern nigeria hospital: a 5-year retrospective cohort study. Ann Med Health Sci Res.

[35] Khusun H, Yip R, Schultink W, Dillon DH (1999). World Health Organization hemoglobin cut-off points for the detection of anemia are valid for an Indonesian population. J Nutr.

[36] Shepherd BE, Blevins M, Vaz LM, Moon TD, Kipp AM, José E (2013). Impact of definitions of loss to follow-up on estimates of retention, disease progression, and mortality: application to an HIV program in Mozambique. Am J Epidemiol.

[37] Chi BH, Yiannoutsos CT, Westfall AO, Newman JE, Zhou J, Cesar C (2011). Universal definition of loss to follow-up in HIV treatment programs: a statistical analysis of 111 facilities in Africa, Asia, and Latin America. PLoS Med.

